# Interleukin-7, a New Cytokine Targeting the Mouse Hypothalamic Arcuate Nucleus: Role in Body Weight and Food Intake Regulation

**DOI:** 10.1371/journal.pone.0009953

**Published:** 2010-04-01

**Authors:** Laurence Macia, Odile Viltart, Myriam Delacre, Christelle Sachot, Laurent Héliot, James P. Di Santo, Isabelle Wolowczuk

**Affiliations:** 1 Univ Lille Nord de France, Lille, France; 2 Laboratory of Neuroimmunoendocrinology and IFR 142, Institut Pasteur de Lille, BP 447, Lille, France; 3 USTL, Inserm U837, JPARC, Development and Plasticity of Postnatal Brain, Lille, France; 4 USTL, Interdisciplinary Research Institute, CNRS USR 3078, Villeneuve d'Ascq, France; 5 Cytokines and Lymphoid Development Unit, Institut Pasteur, Paris, France; 6 Inserm U668, Paris, France; University of Camerino, Italy

## Abstract

Body weight is controlled through peripheral (white adipose tissue) and central (mainly hypothalamus) mechanisms. We have recently obtained evidence that overexpression of interleukin (IL)-7, a critical cytokine involved in lymphopoiesis, can protect against the development of diet-induced obesity in mice. Here we assessed whether IL-7 mediated its effects by modulating hypothalamic function. Acute subcutaneous injection of IL-7 prevented monosodium glutamate-induced obesity, this being correlated with partial protection against cell death in the hypothalamic arcuate nucleus (ARC). Moreover, we showed that IL-7 activated hypothalamic areas involved in regulation of feeding behavior, as indicated by induction of the activation marker c-Fos in neural cells located in the ventromedial part of the ARC and by inhibition of food intake after fasting. Both chains of the IL-7 receptor (IL-7Rα and γ_c_) were expressed in the ARC and IL-7 injection induced STAT-3 phosphorylation in this area. Finally, we established that IL-7 modulated the expression of neuropeptides that tune food intake, with a stimulatory effect on the expression of pro-opiomelanocortin and an inhibitory effect on agouti-related peptide expression in accordance with IL-7 promoting anorectic effects. These results suggest that the immunomodulatory cytokine IL-7 plays an important and unappreciated role in hypothalamic body weight regulation.

## Introduction

Body weight is tightly regulated by complex and intertwined processes involving peripheral tissues, such as the white adipose tissue, as well as the central nervous system, especially the hypothalamus. Alterations of this subtle equilibrium may lead to obesity or lipodystrophy commonly associated with life threatening diseases, like diabetes, insulin-resistance, cardiovascular disorders and some cancers [1].

The hypothalamic arcuate nucleus (ARC) is the master central coordinator of energy homeostasis that adjusts feeding behavior in response to peripheral signals [2], [3]. The ARC contains two major populations of neurons, broadly defined as “anabolic” and “catabolic” neurons. Anabolic neurons co-express the orexigenic neuropeptides agouti-related protein (AgRP) and neuropeptide-Y (NP-Y) [4], upregulation of which promotes weight gain. Catabolic neurons express the anorexigenic neuropeptides cocaine-amphetamine related transcript (CART) [5] or pro-opiomelanocortin (POMC) [6], and are involved in hypophagia and weight loss. The level of expression of these different neuropeptides is finely regulated notably by hormones such as leptin and insulin, both considered as satiety factors [7].

Interestingly, the immune system also actively modulates feeding behavior through a direct hypothalamic effect of the cytokines. Indeed, pro-inflammatory cytokines have been reported to act on the ARC not only during the early phase of the immune response but also under physiological conditions. Thus, interleukin-1β (IL-1β), IL-6 and tumor necrosis factor-α (TNF-α), released by innate immune cells during bacterial infections, modulate feeding behavior [8]. On the other hand, IL-1 receptor antagonist-deficient mice (IL-1Ra^−/−^ mice) are resistant to monosodium glutamate (MSG)-induced obesity [9] while IL-6-deficient mice develop a late onset-obesity [10]. Finally, while IL-18 deficiency leads to obesity, the peripheral injection of IL-18 suppresses appetite [11], [12].

We recently identified IL-7 as a novel cytokine regulating whole-body metabolism, functioning in a fat-to-brain axis (Wolowczuk *et al*., submitted). IL-7 plays a key role in lymphoid homeostasis [13]–[15]. This cytokine is pleiotropic, mostly expressed by bone marrow and thymus stromal cells [16], [17] but also by non-lymphoid cells and tissues such as dermal endothelial cells in skin [18] and abdominal adipose tissue [19]. While IL-7 is mostly known for its potent immune function, we recently identified that mice over-expressing IL-7 were protected from diet-induced obesity associated with decreased food intake (Wolowczuk *et al*., submitted). Remarkably, we further demonstrated that an administration of acute recombinant IL-7 was sufficient to protect mice from gold thioglucose-induced obesity, adipocyte lipid-engulfment and insulin resistance commonly associated with this type of hypothalamic hyperphagic obesity (Wolowczuk *et al*., submitted).

Here we investigate the effects of IL-7 on the hypothalamic areas that participate in the regulation of body weight metabolism. Our results highlight the physiological effects of IL-7 on energy homeostasis and provide evidence for a central role of IL-7 on food intake regulation through a direct effect on ARC cells.

## Results

### IL-7 protects against monosodium glutamate-induced obesity

**Figure 1:**
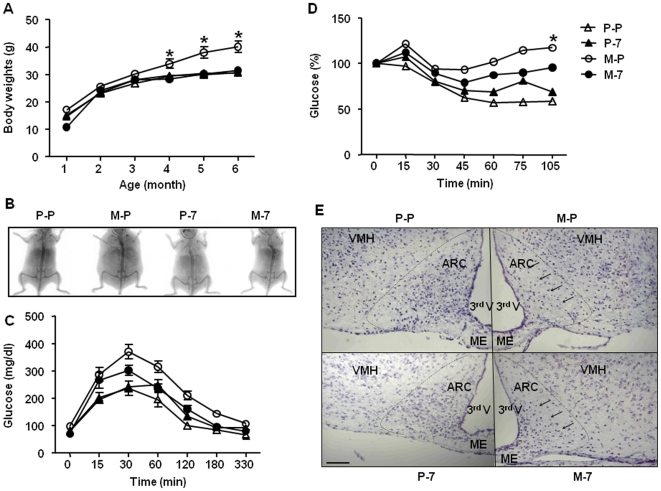
IL-7 protects from MSG-induced body weight gain, increased fat mass, glucose- and insulin-resistance, and arcuate nucleus lesioning. (**A**) Body weight evolution: Mice were injected with PBS (P) from post-natal day 5 (P5) to P12 (group P-P, n = 4;Δ), with monosodium glutamate (MSG) from P5 to P11 then PBS at P12 (group M-P, n = 4;○), with PBS from P5 to P11 then IL-7 at P12 (group P-7, n = 6;▴) or with MSG from P5 to P11 then IL-7 at P12 (group M-7, n = 6;•). Body weights were measured monthly during 6 months. Results are expressed as mean ± SEM. * *p*<0.05. (**B**) Visualization of the fat mass of one representative mice per group using Dual Energy-X-ray Absorptiometry. (**C**) Glucose tolerance test: At the age of 5 months, P-P (Δ), P-7 (▴), M-P (○) and M-7 (•) mice were fasted 18 h before i.p. glucose injection. Glycaemia was measured for each individual animal, from t = 0 to t = 330 min after glucose administration. Results are presented as mean ± SEM. Statistical analysis was performed with a 2-way ANOVA. (**D**) Insulin tolerance test: At the age of 5 months, P-P (Δ), P-7 (▴), M-P (○) and M-7 (•) mice were fasted 4 h before i.p. insulin injection. Glycaemia was measured from t = 0 to t = 105 min after insulin injection. Results are presented as mean ± SEM. Statistical analysis was performed with a 2-way ANOVA. * *p*<0.05. (**E**) Representative microphotographs of frontal hypothalamic sections stained with cresyl violet staining. Brains were harvested from 2-month-old P-P (upper left), P-7 (down left), M-P (upper right) and M-7 (down right) animals. Arrows show the partial cell survival in the M-7-treated group in comparison with the M-P group. Scale bar represents 100 µm. ARC: arcuate nucleus, ME: median eminence, 3^rd^ V: third ventricle, VMH: ventromedial hypothalamic nucleus.

To assess the consequences of IL-7 administration on the development of hypothalamic obesity, we used the well-defined model of administration of monosodium glutamate (MSG), a neurotoxic drug inducing lesions in the hypothalamic arcuate nucleus (ARC) [20]. As expected, MSG-treated mice (M-P group) developed a significant weight gain from 4 month-old (28% increase) compared to mice treated solely either with PBS (P-P group) or IL-7 (P-7 group) (*p*<0.01; Figure 1A). Strikingly, mice that were injected with both MSG and IL-7 (M-7 group) did not show any weight gain (from 1 to 6 months), and displayed a body weight curve similar to that of PBS- or IL-7-treated animals (P-P and P-7 groups, respectively; Figure 1A). Determination of body composition using Dual-Energy X-ray Absorptiometry (Figure 1B) showed that M-P mice had a significant increase of their fat mass (43.5%) compared to PBS- or IL-7-treated animals (16% and 19.3%, respectively). Additionally, IL-7 administration to MSG-treated mice decreased the fat mass back to basal levels (M-7 group, 20.5%), therefore suggesting that the difference of body weight between M-P and M-7 groups (Figure 1A) was likely due to a decrease in fat mass in IL-7-MSG co-treated animals.

Since obesity is often associated with alteration of glucose metabolism, we evaluated *in vivo* glucose tolerance and sensitivity to insulin of mice from the four experimental groups. While glycemia was similar after overnight fasting (respectively for P-P, P-7, M-P and M-7 groups: 82.3±0.5, 79.6±4, 98.5±17.8 and 71.2±10.2 mg/dl), the MSG-treated mice demonstrated an abnormal glucose tolerance test (Figure 1C). Indeed, as early as fifteen minutes after glucose injection, the M-P and M-7 groups showed significant hyperglycaemia compared to P-P mice (M-P *vs* P-P, *p*<0.001; M-7 *vs* P-P, *p*<0.003) and P-7 mice (M-P *vs* P-7, *p*<0.001; M-7 *vs* P-7, *p*<0.001). Thereafter, and for all the time-points assessed, M-P mice had the highest glycaemia (*p*<0.05), whereas mice from the P-P group had the lowest glucose levels (P-P *vs* P-7, *p*<0.05). Interestingly, the M-7 group displayed glucose values between the highest (M-P) and the lowest (P-P) groups, with a slightly delayed return to euglycaemia compared to the M-P group (M-7 *vs* M-P, *p*<0.05; and M-7 *vs* M-P, *p*<0.05). Interestingly, despite the beneficial effects of IL-7 on the MSG treatment, IL-7 alone was responsible for a slowly developing (from 60 minutes after glucose administration) state of a mild, yet significant, glucose intolerance (P-7 *vs* P-P, *p*<0.05). On the other hand, during an insulin tolerance test, the two MSG-treated groups had a significantly reduced response to the hypoglycaemic effects of insulin, when compared with P-P and P-7 groups (*p*<0.05) (Figure 1D), indicating a state of insulin resistance. Interestingly, IL-7 administration to MSG-treated mice (M-7) alleviated the state of insulin-resistance associated with MSG-treatment (M-P) (with *p*<0.001 at t = 15, 30, 45 and 60 minutes), despite being ineffective in restoring the basal reactivity to insulin (with *p*<0.05 at t = 60 between M-7 and P-P).

Since MSG promotes obesity development *via* a neurotoxic effect on the ARC [20], we evaluated if the beneficial effects of IL-7 on MSG treatment were associated with changes in this hypothalamic nucleus (Figure 1E). While MSG treatment induced a specific lesion of the ARC (M-P group), visualized by a drastic loss of cells in this area, the M-7 mice were partially protected from MSG-induced lesions, particularly in the mediobasal region of the ARC, where NPY-expressing neurons are located [21], at eight week-old (Figure 1E) and also at six month-old (data not shown). As expected, P-P and P-7 mice had an intact ARC structure. Altogether, IL-7 protected from obesity and metabolic alterations induced by MSG associated with a neuroprotective effect in the ARC.

### IL-7 prolongs the survival of arcuate nucleus cells in adult mice

**Figure 2:**
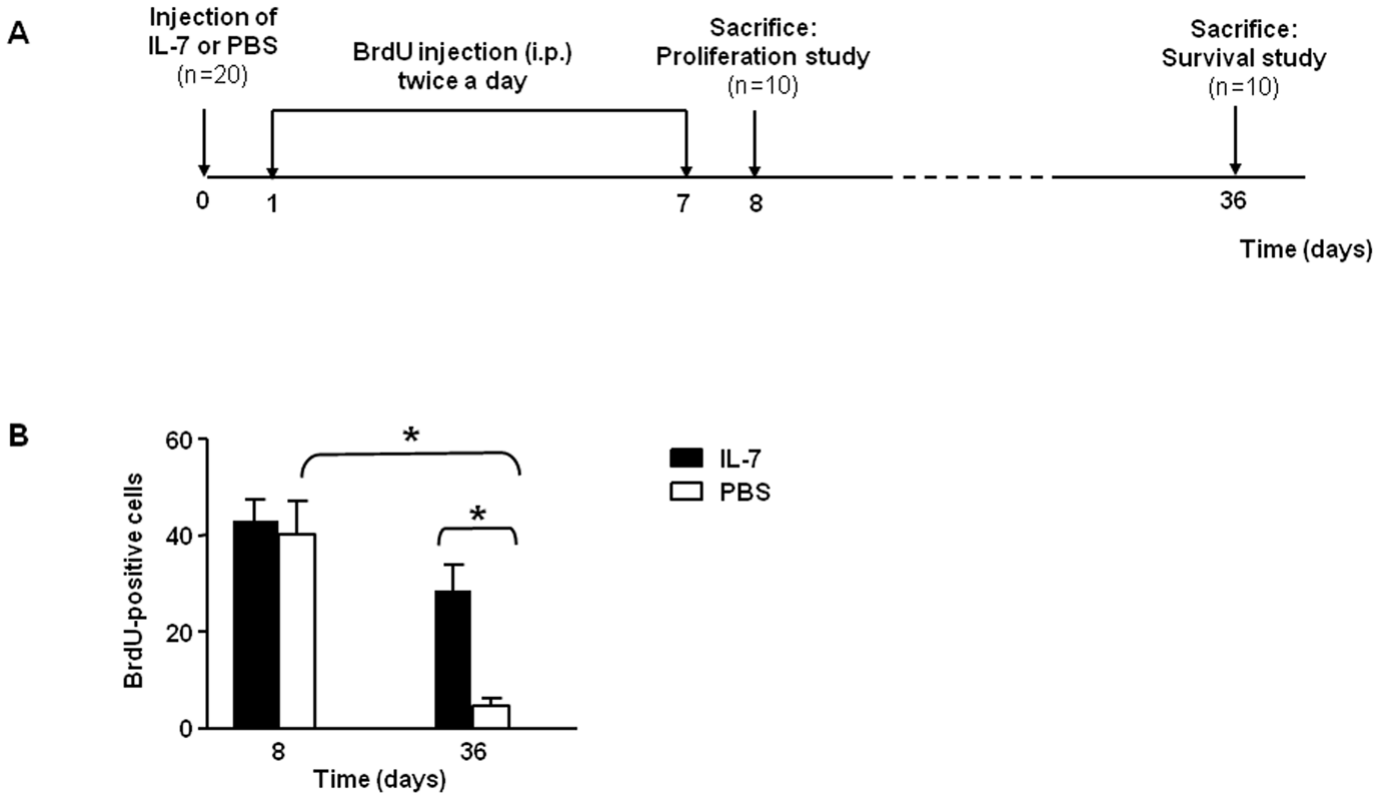
IL-7 improves neural cell-survival. (**A**) Bromodeoxyuridine (BrdU) treatment experimental procedure. (**B**) BrdU positive cells in the ARC of PBS- (□) or IL-7- (▪) treated mice. Animals were sacrificed one day after BrdU treatment (post-natal day 8 (P8)) for neural cell proliferation analysis (n = 5 per group), or 29 days after BrdU treatment (P36) for neural cell survival analysis (n = 5 per group). Data were represented as mean ± SEM of BrdU-positive cells within the ARC per animal and per slice. *p<0.05.

The neuroprotection observed in mice co-treated with MSG and IL-7 suggests that IL-7 promoted either ARC neurons proliferation and/or survival. To understand the mechanisms involved, we compared *in vivo* the effects of IL-7 or PBS treatment on the incorporation of bromodeoxyuridine (BrdU) in mouse hypothalamic cells. The quantification of BrdU positive cells was performed either the day after the end of the BrdU treatment to count the number of newly generated cells in the ARC, or 29 days after the end of the treatment to measure hypothalamic cell-survival rate (Figure 2A). We first observed that IL-7 had no marked effect on the proliferation of cells in the ARC, since the number of BrdU positive cells throughout this area was similar in PBS- and IL-7-treated mice eight days after BrdU injections (Figure 2B). Nevertheless, when analyzing the long-lasting effects of IL-7 injection on cell-survival (*i.e.* 36 days after the first BrdU injections), we observed a reduction of BrdU positive cells in both groups when compared with 8 days post-injection, which is consistent with physiological cell-death. However, we counted more BrdU positive cells in the IL-7-treated mice compared to the control group (*p*<0.05) at this later time point (Figure 2B). These results showed that while IL-7 injection did not affect the ARC cell-proliferation it significantly improved ARC cell-survival.

### IL-7 directly activates arcuate nucleus cells in adult mice

**Figure 3:**
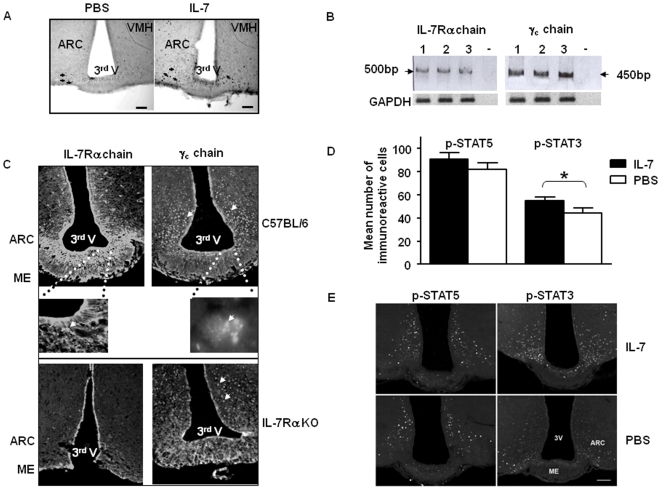
IL-7 activates hypothalamic cells and IL-7 receptor is expressed on hypothalamic cells. (**A**) Representative microphotographs of Fos expression induced by peripheral IL-7 injection in C57BL/6 mice. Fos-immunoreactive cells in arcuate nucleus (ARC) in PBS-treated and IL-7-treated mice. Arrows indicate Fos-imunoreactive cells. 3^rd^ V: third ventricule; VMH: ventromedial hypothalamic nucleus. Scale bars represent 100 µm. (**B**) mRNA expression of IL-7Rα and γ_c_ chains in the whole hypothalamus of 2-month-old C57BL/6 mice (n = 3). Lanes (1, 2, 3) represent one animal. The - lane represents the negative control *i*.*e*. without retro-transcription. (**C**) Representative microphotographs of frontal hypothalamic frozen sections from 2-month-old mice showing fluorescent immunostaining of IL-7Rα and γ_c_ chains in the arcuate nucleus. Frozen section from IL-7Rα KO were used as control for the IL-7Rα specific staining. ARC: arcuate nucleus, ME: median eminence, 3^rd^ V: third ventricule; VMH: ventromedial hypothalamic nucleus. (**D**) Mean number of phosphorylated (p)-STAT5 and p-STAT3 immunoreactive (IR) cells in the hypothalamic arcuate nucleus of C57BL/6 mice injected with recombinant IL-7 (▪; n = 5) or with PBS (□; n = 5). Results are expressed as mean of immunoreactive cells ±SEM per animal and per slice. *p<0.05. (**E**) Representative microphotographs of p-STAT5 and p-STAT3-IR cells in the ARC in PBS-treated and IL-7-treated mice. Scale bar represent 100 µm. ARC: arcuate hypothalamic nucleus; ME: median eminence; 3rd: third ventricle.

These results suggest that the hypothalamus is a central target of IL-7. To identify which hypothalamic areas were targeted, we analyzed c-Fos expression, a marker of early cellular activation, in the ARC of mice injected either with IL-7 or PBS. We found that IL-7 administration led to a significant increase in the number of c-Fos-immunoreactive (Fos-IR) cells in the ARC (11.2±2.4 Fos-IR cells in PBS-treated mice and 20.2±2.7 Fos-IR cells in IL-7-treated mice, *p*<0.001). Interestingly, the distribution of Fos-IR cells was confined to the ventromedial part of the ARC (Figure 3A), coincident with the specific areas protected by IL-7 from MSG-induced lesion (Figure 1E).

To determine if the activation of ARC neurons by peripheral IL-7 injection was mediated *via* a direct effect, we studied the expression of IL-7 receptor, composed of two chains: IL-7Rα and γ_c_ [22]-[24], both at the transcriptional and translational levels. Both IL-7Rα and γ_c_ chains mRNAs were expressed in isolated hypothalamus from wild-type mouse brain (Figure 3B) and were localized in the ARC as visualized by immunohistology (Figure 3C). While γ_c_ was broadly distributed in the ARC, the expression of IL-7Rα was restricted to the ventromedial part of the ARC and the median eminence.

In the immune cells, IL-7R signaling results in robust STAT5A/B phosphorylation and, to a lesser extent, in STAT3 phosphorylation [25], [26]. To further investigate the mechanism of IL-7-driven activation of arcuate nucleus cells, we analyzed the expression of phosphorylated (p)-STAT5 and p-STAT3 in the arcuate nucleus of IL-7-injected wild-type mice. While IL-7 treatment did not modify STAT5 phosphorylation (81.8±5.7 p-STAT5-IR cells in PBS-treated mice and 90.6±5.9 p-STAT5-IR cells in IL-7-treated mice), it significantly stimulated STAT3 phosphorylation (44.4±4.3 p-STAT3-IR cells in PBS-treated mice and 54.9±3.2 in IL-7-treated mice, *p*<0.05) (Figure 3D).

We found a homogeneous distribution of phosphorylated STAT5 in the ARC of both groups, but the IL-7-treated animals exhibited more immunoreactive cells in the ventromedial ARC, where NP-Y/AgRP neurons are located (Figure 3E). More caudally, p-STAT5-IR cells were detected in the third ventricle and were located in the dorsal part of the ARC in both groups (data not shown). On the other hand, we detected STAT3-IR cells specifically in the ventromedial ARC, all along the ARC, with an overall significant higher number in the IL-7-treated mice. Interestingly, we found a stronger labelling of p-STAT5 positive fibers on the external part of the median eminence compare to p-STAT3 (Figure 3E), area where we described the expression of IL-7Rα (Figure 3C). These results show that both IL-7R chains were present in the ARC and that IL-7 treatment activated the ARC nucleus cells, correlated with the induction of STAT3 phosphorylation.

### IL-7 modulates the expression of hypothalamic neuropeptides in adult mice

**Figure 4:**
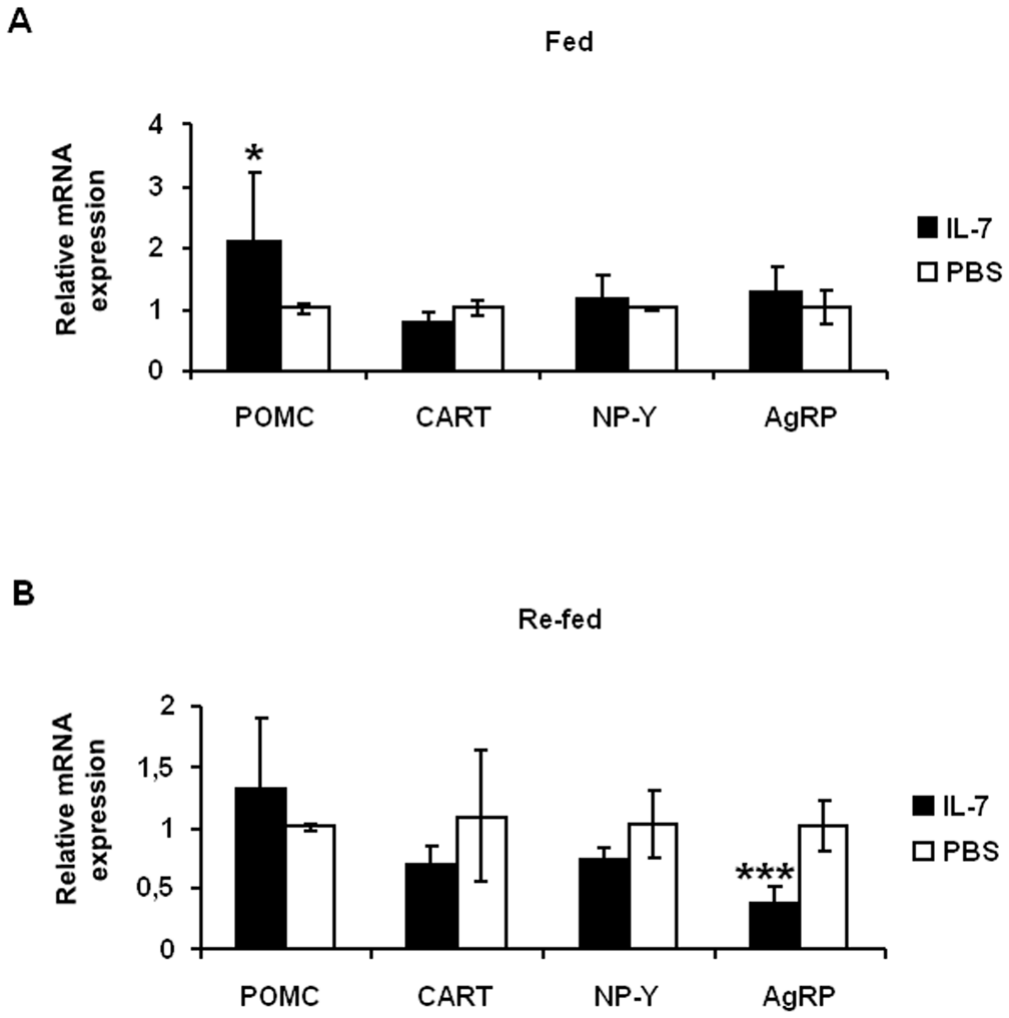
IL-7 modulates hypothalamic neuropeptide mRNA gene expression. PBS ( = 5, □) or IL-7 (n = 5, ▪) was i.p. injected in 2-month-old C57BL/6 mice. The expression of hypothalamic neuropeptides was assessed by real-time PCR, in fed (**A**) or in re-fed (**B**) condition. The effects of IL-7 treatment were evaluated by calculating the relative expression levels as follows: 2^ΔCt^ (ΔCt = mean Ct genes of interest - mean Ct GAPDH), using the raw cycle-threshold (Ct) values. *p<0.05, *** *p*<0.001. POMC: pro-opiomelanocortin, CART: cocaine-amphetamine related peptide, NP-Y: neuropeptide-Y, AgRP: Agouti-related peptide.

Subpopulations of neurons located in the ventromedial part of the ARC express key neuropeptides controlling feeding behavior. To determine if IL-7 modulated these neuropeptides expressions and thus potentially food intake, we quantified the expression of POMC and CART (anorexigenic peptides) and of NP-Y and AgRP (orexigenic peptides) in the hypothalamus of IL-7- or PBS-treated mice either fed *ad libitum* or re-fed after an overnight fasting. Under fed conditions, while IL-7 treatment had no effect on AgRP, CART and NP-Y mRNA expression, it induced a significant increase (87%, *p*<0.05) in POMC mRNA expression (Figure 4A). Moreover, after four hours of re-feeding after fasting conditions, while POMC, CART and NPY had comparable level of expressions between IL-7 and PBS-treated mice, the expression of AgRP was significantly inhibited after IL-7 treatment (66%, *p*<0.05) (Figure 4B). These results show a regulatory effect of IL-7 on ARC neuropeptides by stimulating POMC under fed conditions and inhibiting AgRP under fasted re-fed conditions.

### IL-7 inhibits food intake in re-feeding conditions in adult mice

**Figure 5:**
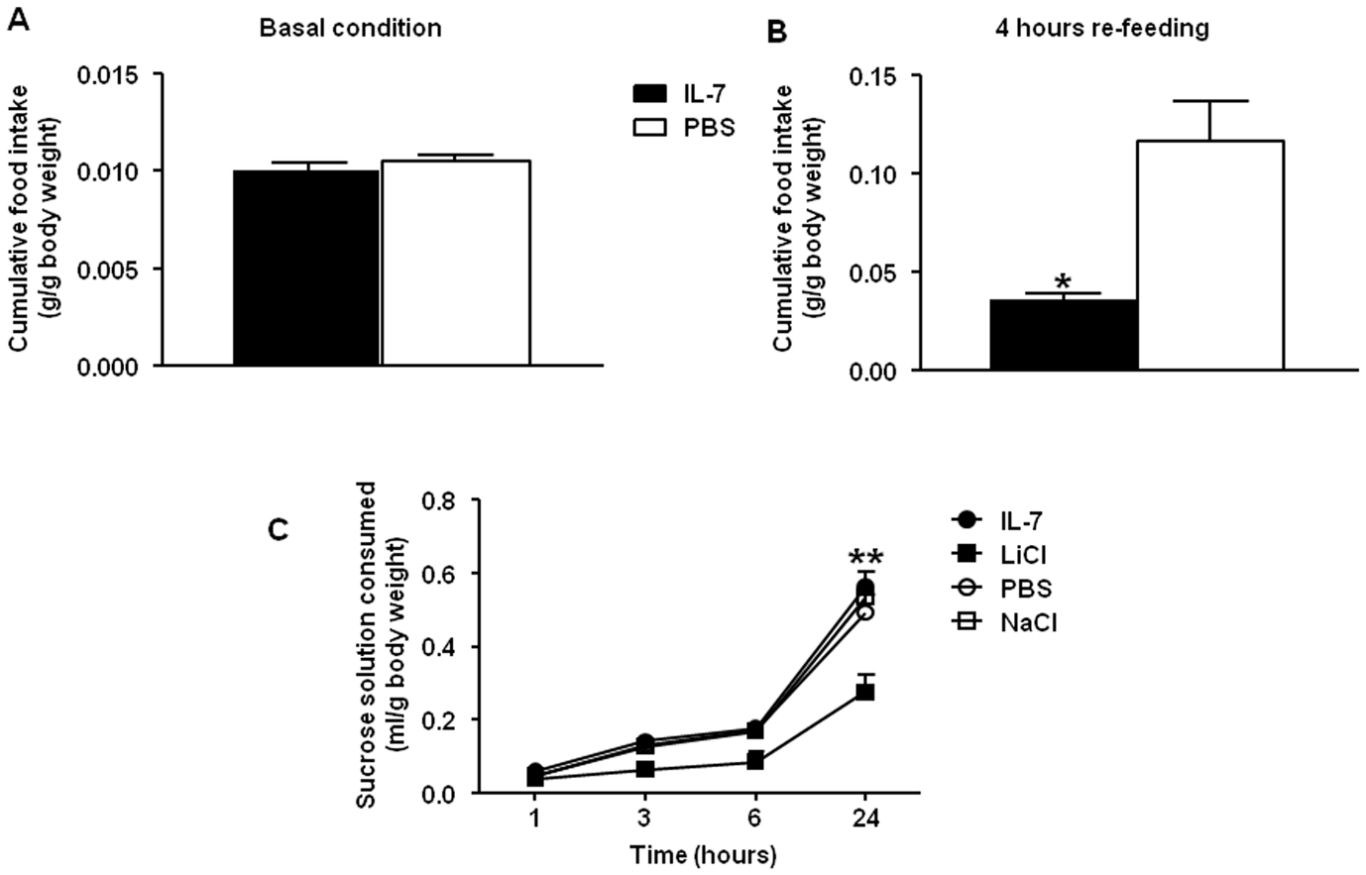
IL-7 regulates food intake after fasting. (**A**) Food intake during basal conditions in IL-7- (▪) or PBS- (□) treated C57BL/6 mice (n = 7 per group). Results are expressed as the mean ± SEM of cumulative food intake in g/g of body weight. (**B**) Animals were overnight-fasted, treated with IL-7 (▪) or PBS (□) before getting free access to food (n = 5 per group). Food intake was measured during the 5 h following re-access to food and was expressed as mean ± SEM of cumulative food intake in g/g of body weight. *p<0.05. (**C**) *Prior* to the taste aversion assay, water was removed from animal cages overnight. Taste solution of 5% sucrose was offered for 30 min and mice were either s.c. injected with 0.3 µg of IL-7 (n = 5,•) or PBS as control (n = 4,○), or i.p. injected with 6 mEq of LiCl (n = 5,▪) or NaCl as control (n = 4,□). After injection, animals got free access to water containing 0.5% of sucrose. Sucrose cumulative consumption was individually measured 1 h, 3 h, 6 h and 24 h after injection. Results are expressed as mean of sucrose consumption in ml/body weight in g ± SEM. *p<0.05.

To investigate if the changes induced by IL-7 on the ARC neuropeptide expression modulated feeding behavior, we measured the effects of IL-7 treatment on basal food intake and on re-feeding after fasting. While food consumption was similar between IL-7 and PBS-treated mice under fed conditions (Figure 5A), we observed that after an overnight-fast, IL-7 treatment significantly reduced the food intake of mice for the first four hours post re-feeding (Figure 5B). Moreover, the inhibitory effect of IL-7 on food intake lasted for 24 hours before return to basal food intake (data not shown). Importantly, to test whether the observed reducing effects of IL-7 on food intake might rely on visceral illness, we performed a taste aversion assay comparing the effects of IL-7 on sucrose consumption to lithium chloride (LiCl) effects, as previously described [27]. We found that after an overnight deprivation of water and one hour of habituation to a solution of sucrose, mice treated with IL-7, PBS or saline consumed similar amounts of sucrose 24 hours post injection (Figure 5C). On the other hand, in the same experimental procedure, LiCl-treated mice developed a taste aversion with a significant reduction of sucrose consumption (Figure 5C). These results suggest that IL-7-mediated inhibition of food intake is not secondary to taste aversion.

## Discussion

In this report, we provide evidence that IL-7 targets hypothalamic brain areas to regulate body weight and feeding behavior. IL-7 not only protected from obesity development but also reduced food intake by directly targeting the hypothalamus, and more precisely the ARC. This work and our recent description of a new role of IL-7 in the regulation of energy homeostasis (Wolowczuk *et al*. submitted) identify the novel role for this cytokine in body weight and metabolic regulation.

Interestingly, while as previously described, MSG treatment was associated to significant neural cell-loss in the ARC leading to the obesity development, a single injection of IL-7 completely protected the mice from gaining weight. Moreover, IL-7 treatment also greatly improved obesity-associated disorders with a complete restoration of insulin sensitivity, and a partial improvement of the glucose tolerance in adulthood. These metabolic changes were associated with a partial protection of the mediobasal part of the ARC, where are located NP-Y and somatostatin neurons [23], underlying a potential central role of IL-7. This protection was correlated with the pro-survival effects of IL-7 on neural cells since we showed that a single injection of IL-7 was sufficient to increase neural cell-survival without affecting their proliferation rate. In accordance with its pro-survival effects on lymphocytes [15], IL-7 might exert its neural protective role through the up-regulation of the anti-apoptotic factor bcl-2, and thus counteract or compensate for bcl-2 down-regulation in MSG-induced apoptosis [28]. However, despite this central effect, a peripheral role of IL-7 in the protection against MSG-induced obesity and metabolic alterations could not be excluded. Indeed, a protective effect by targeting the white adipose tissue was previously described with the pro-inflammatory cytokine IL-1 [9] and our previous report showed the critical role of IL-7 on white adipose tissue (Wolowczuk *et al*., submitted).

Here we demonstrate that IL-7 has a central hypothalamic effect. Indeed, as mentioned earlier, a single injection of IL-7 was sufficient to trigger neuronal activation in the adult mice ARC, but we also showed that IL-7 induced the expression of the early activation marker c-Fos in the ventromedial area of the ARC. As c-Fos activation might involve multisynaptic neuronal relay [29], its expression can be due to a direct but also by an indirect effect of IL-7. However, the direct effect was strengthened by our demonstration of the presence of both chains of the IL-7 receptor in this area, respectively the IL-7Rα and the γ_c_ chains, distributed in the ventromedial part of the ARC and in the median eminence. IL-7 signaling during B and T lymphopoiesis involves primarily STAT5 phosphorylation [25], [30], [31], although a specific role for STAT3 phosphorylation has been reported in mediating early B-cell progenitor survival [32]. Based on the knowledge that IL-7R (our results) and STAT5 [33] are present in hypothalamic neurons, we asked whether STAT5A/B and/or STAT3 contributed to IL-7-mediated regulation of hypothalamic arcuate nucleus cells. Interestingly, we showed that while IL-7 administration did not modify STAT5 activation when compared with PBS-treated animals, it significantly increased the number of p-STAT3-IR cells. Although IL-7R signaling is known to induce STAT1, STAT3 and STAT5 phosphorylation [25], [26], our results suggest that STAT3 plays the dominant role in IL-7-mediated arcuate nucleus neuronal activation and/or survival. This result is similar to the reported effects of leptin in hypothalamic neurons in which it induced STAT3 phosphorylation through leptin receptor prior to c-Fos induced expression [34]. Strikingly, IL-7-treatment induced the expression of c-Fos in the ventromedial part of the ARC, close to the third ventricle, precisely where IL-7 induced p-STAT3 activated cells were located. The p-STAT5 staining was more homogeneously distributed in this area, with a highest number of p-STAT5-IR cells located caudally in the dorsal ARC. Altogether, this further supported our demonstration of a preferential activation of STAT3 in IL-7-responsive hypothalamic cells. Moreover, few p-STAT5-IR cells were also detected in paraventricular hypothalamus, periventricular nuclei and lateral hypothalamus, areas where anorexigenic and orexigenic neurons were found (data not shown).

Since the ARC modulates body weight and feeding behavior through the differential expression of anorexigenic and orexigenic neuropeptides, we further investigated the central effect of IL-7 on these key hypothalamic neuropeptides in adult mice. In condition of *ad libitum* feeding with a normal chow diet, we found that a single administration of IL-7 in mice drastically increased the expression of hypothalamic POMC, a major anorexigenic neuropeptide [35] without affecting food intake. However, we show that under re-feeding conditions after fasting, IL-7 treatment significantly decreased mice food intake showing the potent anorectic effect of this cytokine. Like IL-7, the mammalian target of rapamycin (mTOR) is an anti-apoptotic factor promoting cell-growth [36], [37] and regulates food intake [38]. Thus, as previously reported in lymphocytes, IL-7 might mediate its central effect on neuronal cell-survival and on feeding behavior *via* mTOR. Interestingly, this inhibitory effect of IL-7 on food intake was correlated with a specific inhibition of AgRP as described in a transactivation-deficient FoxO1 (Forkhead box containing protein, O subfamily1) mutant mice [39], with no change of expression of the other key neuropeptides studied. Importantly, contrary to other cytokines described to induce anorexia secondary to nausea [8], we demonstrate that this modulation of feeding is mediated by IL-7 *per se* and not by sickness.

In conclusion, we show here for the first time the potent central role of IL-7 on energy regulation by directly signaling in the ARC, key hypothalamic area controlling feeding behavior and metabolism. Our initial work, in which we used a model of IL-7 overexpressing mice [40], revealed a novel aspect of IL-7 biology, namely its role in the regulation of whole-body metabolism (Wolowczuk et al., submitted). Indeed, beside defective white adipose tissue formation and functionality in IL-7 transgenic animals, we also observed evidence of IL-7's central action. The present work furthered these initial observations, notably regarding the effects of IL-7 on the hypothalamus. Altogether, our results suggest that IL-7 acts as a novel key regulator of food intake under specific conditions like re-feeding after fasting (present paper), feeding with sucrose-enriched regimen (Viltart *et al*., manuscript in preparation) or feeding with a high-fat diet (Wolowczuk *et al*., submitted). We thus propose IL-7 as a new essential factor participating in the complex integration of peripheral hormonal-immune signals in the central nervous system to control body weight.

Our work thereby opens a wide avenue for identifying novel targets to improve the existing treatment and/or to develop new treatments for obesity and/or appetite disorders.

## Materials and Methods

### Animals

Adult female and male mice C57BL/6J@Rj (8–12 week-old; Janvier Laboratory, Le Genest-St-Isle, France) were used. For the monosodium glutamate experiments, newborn mice from our breeding colonies were utilized. Mice were housed in a pathogen-free area in our animal facilities and maintained in a temperature- (20±2°C) and humidity- (60%) controlled room on a daily cycle of 12 h light and darkness. Animals had *ad libitum* access to water and food (standard chow diet, U.A.R., Epinay s/Orge, France) unless indicated.

### Ethics statement

Experiments were done according to the institutional ethical guidelines for laboratory animal care (European Communities Council Directive of 1986, 86/609/EEC) and approved by the Departmental Direction of Veterinary Services (Prefecture of Lille, France; authorization number: 59–350152).

### Monosodium glutamate and IL-7 treatments

Neurotoxic lesion of hypothalamic arcuate nucleus was performed by subcutaneous (s.c.) injection of monosodium glutamate (MSG; 2 mg/g body weight/day; Sigma, L'Isle d'Abeau Chesnes, France) in neonate mice. The following experimental groups were designed: The M-P group concerned mice (n = 4) s.c. injected with MSG from postnatal days 5 to 11 (P5 to P11), then with PBS at P12. The M-7 group (n = 6) received MSG from P5 to P11, then were s.c. injected with murine recombinant IL-7 (0.3 µg/mouse; Peprotech, London, UK) at P12. The P-P group (n = 4) was s.c. treated by PBS from P5 to P12. The P-7 group (n = 6) received PBS from P5 to P11, before receiving IL-7 (0.3 µg/mouse) on P12. Body weights were individually recorded weekly during six months. Glucose and insulin tolerance testing were done 5 months post-MSG treatment with a two-week interval between either testing. Glucose (i.p. injection, 2 g/kg body weight, D-glucose; Sigma) tolerance test was performed in overnight-fasted mice. Blood samples were obtained from the tail vein at 0, 15, 30, 60, 120, 180 and 330 minutes. Insulin (i.p. injection, 0.75 mU/g body weight, human insulin; Novo Nordisk Pharmaceutique S.A., Boulogne-Billancourt, France) tolerance test was performed on 4 h-fasted mice. Blood was collected from the tail vein before injection (time 0) and 15, 30, 45, 60, 75 and 105 minutes after insulin administration.Glucose levels were measured using glucose strips and an automatic glucometer (Glucotrend®, Roche Diagnostics, Meylan, France).

At the end of the experiment, one representative mouse of each group was sacrificed by cervical dislocation and percentage of fat mass was determined by Dual-Energy X-ray Absorptiometry (DXA) (PIXIMUS™, LUNAR, Lambesc, France). The experiment was performed twice from independent breeding.

### Histological analysis of the hypothalamus

Mice were deeply anesthetized with 5% pentobarbital (Ceva Santé Animale, Libourne, France) and perfused transcardially with 0.9% NaCl followed by a solution of PBS containing 4% paraformaldehyde (PFA). Brains were dissected and post-fixed in PFA 4% at 4°C for two days. Then, brains were cryo-protected by immersion in a 20% sucrose solution, frozen in dry ice and stored at −80°C before being further sectioned with a cryostat (Leica microsystem, Nussloch, Germany). Hypothalamus frozen sections of 12-µm thickness were serially collected onto gelatin-alum-chrome-coated slides. Hypothalamus was delimited from Bregma −1.22 mm to −2.70 mm [41]. To visualize the lesions induced by MSG in the adult hypothalamus, cresyl violet staining was performed. Slices were then dehydrated in graded alcohols, cleared in xylene and coverslipped with Eukitt (Poly Labo, Strasbourg, France).

### *In vivo* Bromodeoxyuridin (BrdU) incorporation assay

In another set of experiments, 8-week-old mice (n = 10 per group) were s.c. injected with IL-7 (0.3 µg per mouse; Peprotech) or with PBS, at day 0. From day 1 to day 7, mice received i.p. injections of BrdU (25 mg/g body weight, Sigma, Steinheim, Germany), diluted in 0.9% NaCl, twice a day every 8 hours. At day 8, half of the mice were sacrificed, by transcardial perfusion as described above (for the proliferation study) and the remaining half were sacrificed at day 36 (for the survival study). After 4% PFA post-fixation, brains were cut with a vibratome (Leica microsystem) in serial free-floating coronal sections of 35-µm thickness. Slices were collected in 0.1% azide phosphate buffer before being stored at 4°C. One section on two was kept onto the brain.

To reveal BrdU *in vivo* incorporation, the free-floating hypothalamic slices were first incubated in HCl 2N at 37°C for 30 min and then transferred in 0.1M borate buffer (pH 8.5) for 10 min, at room temperature. Slices were incubated for 24 hours with a rat monoclonal IgG anti-BrdU (1800; Abcys, Paris, France) and then, the secondary biotinylated polyclonal goat anti-rat (Fab')_2_ antiserum (1∶500; Jackson ImmunoResearch, West Grove, PA, USA) was applied for 2 h in PBS containing 0.1% Triton®X-100. Sections were further revealed using the 3-3′ di-aminobenzidine (DAB) glucose oxidase protocol [42] and mounted on gelatine-alum-chrome-coated slides, dehydrated, cleared in xylene and coverslipped with Eukitt. Finally, BrdU positive cells were counted bilaterally throughout the hypothalamic arcuate nucleus on a photonic microscope (Axioskop50, Zeiss, Oberkochen, Germany).

### Immunohistochemical detection of c-Fos protein, p-STAT3 and p-STAT5 in the hypothalamus

Eight week-old mice were s.c. injected with IL-7 (0.3 µg per mouse; Peprotech) or with PBS (n = 6 in each group). Two hours after injection, animals were anesthetized with 5% pentobarbital sodium and were sacrificed. The hypothalamus were removed and free-floating sections were prepared as described above. Coronal 35-µm thick hypothalamic sections were incubated 48 h with an anti-*c-Fos* rabbit polyclonal IgG (sc-52, 1∶10,000; Santa Cruz Biotechnology, Santa Cruz, MA, USA), as primary antibody, before a further 2 h-incubation with biotinylated polyclonal donkey anti-rabbit (Fab')_2_ (1∶500 Jackson Immunoresearch, Immunotech, Marseille, France), used as secondary antibody. Finally, the immunolabelling was revealed using the DAB glucose oxidase protocol [42]. The Fos-immunoreactive cells were bilaterally counted in the arcuate nucleus and in the suprachiasmatic area as control, using a photonic microcoscope (Zeiss, Germany).

In another set of experiments, 8-week-old mice were s.c. injected with IL-7 (0.3 µg per mouse; Peprotech) or with PBS (n = 5 in each group). Forty minutes after injection, animals were anesthetized with 5% pentobarbital sodium and then rapidly perfused transcardially as described above. Brains were removed, post-fixed for 2 h in the same fixative at 4°C, then sunk in a solution of 0.02M K^+^ in PBS (KPBS) with 20% sucrose at 4°C before to be frozen in 2 methylbutane cooled to −50°C with dry ice. Coronal 35-µm thick hypothalamic sections were performed using a cryostat. Immunochemistry was carried out by slight modifications of the method described by Hosoi *et al.* [43]. Unless noticed, all the following steps were done at room temperature. Several rinses with 0.02M KPBS were performed between each step. Firstly, slices were pre-treated for 20 min in a solution of 5% NaOH and 0.5% H_2_O_2_ in 0.02M KPBS before being incubated for 10 min in a solution of 0.3% glycine in 0.02M KPBS. The sections were secondly placed in 0.03% SDS for 10 min before a 20 min incubation in a solution of 0.02M KPBS containing 4% normal goat serum (Sigma), 0.4% Triton®X-100 and 1% BSA. Finally, the sections were incubated for 48 h at 4°C with rabbit monoclonal antibody anti-phosphorylated STAT5 (Tyr694, Cell Signaling Technology #9359, Danvers, MA, USA) or rabbit monoclonal antibody anti-phosphorylated STAT3 (Tyr705, Cell Signaling Technology #9131) respectively diluted at 1∶200 and 1∶1000 in 1% normal goat serum, 0.4% Triton®X-100 and 1% BSA. The immunolabeling was revealed using a goat anti-rabbit Alexa568-conjugated secondary antibody (1∶200; Jackson Immunoresearch, Immunotech) in 1% normal goat serum and 0.3% Triton®X-100 for 2 h. Sections were counterstained with Hoescht vital staining (2%; In Vitrogen, Cergy-Pontoise, France) for 3 min. Slides were coverslipped with buffered glycerol mounting medium (Sigma). Immunoreactive cells were bilaterally enumerated in the arcuate nucleus using a fluorescent microscope equipped with an apotome (Zeiss) and the ImageJ analysis system (NIH Image, National Center for Biotechnology Information). The average number of cells per section and per mice was taken for statistical comparisons.

### Immunohistochemistry for IL-7 receptor detection in the hypothalamus

The presence of the IL-7 receptor in the hypothalamus was assessed by fluorescent microscopy (Leica microsystem). Briefly, 4 mice were transcardially perfused with 4% PFA as previously described. After post-fixation, cryoprotection in 20% sucrose and freezing in dry ice, 12 µm-thick coronal frozen sections were obtained with a cryostat. Sections were saturated 30 min with PBS-3% BSA and incubated overnight with the following primary antibodies: rabbit polyclonal IgG anti-murine IL-7Rα (1∶50; Santa Cruz Biotechnology) and rabbit polyclonal IgG anti-IL-2Rγ (1∶50; Santa Cruz Biotechnology). The secondary antibody used was a donkey anti-rabbit coupled with Cy™2-conjugated F(ab')_2_ (1∶500; Jackson ImmunoResearch, Cambridge, UK) incubated during 90 minutes. At each step, 0.1% Triton®X-100 was used for membrane permeabilization. Slides were mounted using Immumount (Thermo Shandon, Pittsburgh, PA, USA). Mice genetically deficient for the expression of IL-7Rα chain (IL-7Rα KO) [44] were used as negative control.

### PCR and quantitative real-time PCR analysis

To analyse the expression of both chains of the IL-7 receptor in the hypothalamus (α and γ_c_ chains), eight-week-old mice (n = 3) were sacrificed by cervical dislocation. The hypothalamus were carefully dissected and immediately frozen in liquid nitrogen before being homogenized and extracted in TRIzol® (Invitrogen, Paisley, Scotland). One µg of total RNA was reverse-transcribed and amplified using the Qbiotaq polymerase (Q Biogen, Illkirch, France). The housekeeping gene Glyceraldehyde-3-phosphate dehydrogenase (GAPDH) was used as control. To test whether IL-7 could modulate the expression of hypothalamic neuropeptides, eight-week-old mice (n = 5 per group) were s.c. injected with IL-7 (0.3 µg per mouse, Peprotech) or with PBS. Animals were sacrificed 4 h later, the hypothalamuses were harvested and RNA extraction and reverse transcription were performed as described above. The resulting cDNA was used as template for real-time quantitative PCR using a LightCycler (Roche). Results were normalized to the expression of the GAPDH. All results of quantitative PCR (Q-PCR) are expressed as relative mRNA expression levels determined using a method (2^ΔCt^; Ct = Cycle threshold) described previously [45]. Primer sequences used are indicated in Table 1.

**Table 1:** Primer sequences used for real-time PCR.

Gene	Forward 5′-3′	Reverse 5′-3′
GAPDH	TGCCCAGAACATCATCCTG	TCAGATCCACGACGGACACA
IL-7Rα	GCCGAGGCTCCCTCTGACCTGAAAG	GGGGAGACTAGGCCATACGACAGG
AgrP	GCATCAGAAGGCCTGACC	TCGCGGTTCTGTGGATCT
NP-Y	GCTTGAAGACCCTTCCATTGGTG	GGCGGAGTCCAGCCTAGTGG
POMC	TGGTGCCTGGAGAGCAGCCACTGC	TGGAGTAGGAGCGCTTGCCCTCG
CART	CGAGAAGAAGTACGGCCAAG	GGAATATGGGAACCGAAGGT
γ_c_	CTCCCTGCCTAGTGTGGATGA	CACTGTAGTCTGGCTGCAGCA

### Feeding behavior

First, to test the effect of IL-7 on basal food intake, mice were s.c. injected with IL-7 (0.3 µg, Peprotech) or PBS (n = 7 per group), and cumulative food intake was measured each hour of a 6-h period. Concerning the re-feeding conditions, animals were overnight-fasted (with free access to water) before being s.c. injected with IL-7 (0.3 µg, Peprotech) or PBS (n = 5 per group). Ten minutes after injection, mice were given *ad libitum* access to food. Food intake was measured every hour during 5 hours and 24 h later.For the determination of mRNA expression levels of hypothalamic neuropeptides (described above), mice were sacrificed by cervical dislocation 4 h after IL-7 injection (fed condition) or 4 h after the re-feeding period (re-fed condition) (n = 5 per group).

### Taste aversion assay

On the experimental day, overnight water-deprived mice were given access to a 5% sucrose solution for 30 min. At the end of the 30 min access to sucrose, mice were divided in the following four groups: mice (n = 5) i.p. injected with a solution of lithium chloride (LiCl, Sigma-Aldrich, St Quentin Fallavier) diluted in NaCl 0.9% and administrated at the concentration of 6 mEq/kg in a final volume of 500 µl, an optimal concentration described elsewhere [36]; mice (n = 4) s.c. injected with 500 µl of NaCl 0.9%; mice (n = 5) s.c. injected with 0.3 µg of IL-7 diluted in 30 µl of PBS; mice (n = 4) s.c. injected with 30 µl of PBS. Animals from each group got then free access to a sucrose 5% solution. Sucrose consumption was measured before treatment, 3 h, 6 h, and 24 h after injections.

### Statistical analysis

All data were expressed as mean ± SEM. Comparisons were made using the Student's t test or Mann Whitney test when the normality test failed, and a two-way ANOVA followed by the Tukey *post hoc* test, when appropriate. *p* values less than 0.05 were considered statistically significant.
